# Serological investigation of Gyrovirus homsa1 infections in chickens in China

**DOI:** 10.1186/s12917-022-03334-0

**Published:** 2022-06-18

**Authors:** Shicheng Zhang, Shiyu Yuan, Tianxing Yan, Gen Li, Xiaojing Hao, Defang Zhou, Ruiqi Li, Yubao Li, Ziqiang Cheng

**Affiliations:** 1 College of Veterinary Medicine, Shandong Agricultural University, No.61, Daizong Street, Taian, 271018 China; 2 College of Veterinary Medicine, Qingdao Agricultural University, Qingdao, 266000 China; 3 Qingdao Animal Husbandry Workstation (Qingdao Animal Husbandry and Veterinary Research Institute), Qingdao, 266000 China; 4 Agricultural Science and Engineering School, Liaocheng University, Liaocheng, 252000 China

**Keywords:** Gyrovirus homsa1, Seroepidemiology, Indirect ELISA, Chicken

## Abstract

**Background:**

Gyrovirus homsa1 (GyH1) (also known as Gyrovirus 3, GyV3) is a non-enveloped, small, single-stranded DNA virus, which was first identified in children with acute diarrhea, and was subsequently detected in marketed chickens, broilers with transmissible viral proventriculitis (TVP), and mammals. GyH1 is a pathogenic virus in chickens, causing aplastic anemia, immunosuppression, and multisystem damage. However, the seroepidemiology of GyH1 infection in chickens remains unclear. Here, we investigated the seroprevalence of GyH1 in chickens by ELISA to reveal the endemic status of GyH1 in China.

**Results:**

An indirect ELISA with high sensitivity and specificity was developed for investigation of seroepidemiology of GyH1 in chickens in China. The seropositive rate of GyH1 ranged from 0.6% to 7.7% in thirteen provinces, and ranged from 4.1% to 8.1% in eight species chickens. The seropositive rate of GyH1 in broiler breeders was significantly higher than that of in layers. There was a negative correlation between seropositive rate and age of chickens. The highest and lowest seropositive rate were present in chickens at 30–60 days and over 180 days, respectively.

**Conclusions:**

The seroepidemiological investigation results demonstrated that natural GyH1 infection is widespread in chickens in China. Different species showed different susceptibility for GyH1. Aged chickens showed obvious age-resistance to GyH1. GyH1 has shown a high risk to the poultry industry and should be highly concerned.

**Supplementary Information:**

The online version contains supplementary material available at 10.1186/s12917-022-03334-0.

## Background

Gyroviruses (GyVs) are a group of icosahedral, small, non-enveloped viruses, consisting of 2.0–2.5 kb single-stranded circular and negative DNA genomes. GyVs were initially reassigned to the *Anelloviridae* family from the *Gyrovirus* genus of the family *Circoviridae* in 2017, as GyVs were structurally and genetically unrelated to other members of the family *Circoviridae* [1]. Recently, numerous *Gyroviruses* have been identified in multiple hosts by viral metagenomics [2–13]. Interestingly, the pathogenic mechanism of these GyVs remains obscure, although genetic and structural similarities between animal and human sequences have suggested their zoonotic potential [14].

Gyrovirus homsa1 (GyH1) (also known as Gyrovirus 3, GyV3), a member of GyVs, was first detected in patients with acute childhood gastroenteritis in Chile [4]. It was then detected in chicken sold on the market [7], broilers with transmissible viral proventriculitis (TVP) in China [15], apparently healthy wild birds in Brazil [16], and diarrhea cats in China [17]. The wide distribution of multiple variability, temporality and spatiality of GyH1 suggests the possible pathogenesis. Previous studies have shown that GyH1 can independently infect chickens, causing immunosuppression, aplastic anemia and TVP [15]. Moreover, it can infect mammals cross-species, causing aplastic anemia and immunosuppression [18], suggesting the transmission of GyH1 may be at risk of human infection. Previous investigation by PCR showed that the prevalence rate of GyH1 was 12.5% in China [15], while serological investigation has not been conducted.

The GyH1 genome contains three partially overlapping open reading frames (ORFs) encoding three viral proteins, the capsid protein VP1, the scaffolding protein VP2, the apoptin protein VP3 [2]. Apparently, VP1 is the only structural protein that plays an important role in virus virulence, assembly, replication, and infection. Interestingly, it can interact with the VP2 protein to induce the production of neutralizing antibodies [18–20]. Therefore, we established the enzyme linked immunosorbent assay (ELISA) based on capsid proteins for anti-GyH1 antibody detection in this study.

GyH1 infection of chickens can result in severe economic losses to chicken farms and is a known cause of immunosuppression, aplastic anemia, TVP and multisystem damage. However, the prevalence of GyH1 infection in commercial chickens in China remains unknown. Here, we developed an indirect ELISA for the detection of GyH1-specific antibodies to reveal the prevalence of GyH1 in commercial chickens in China. The results of this study will contribute to the prevention and control of GyH1.

## Results

### Expression, purification and verification of VP1 proteins

**Fig. 1:**
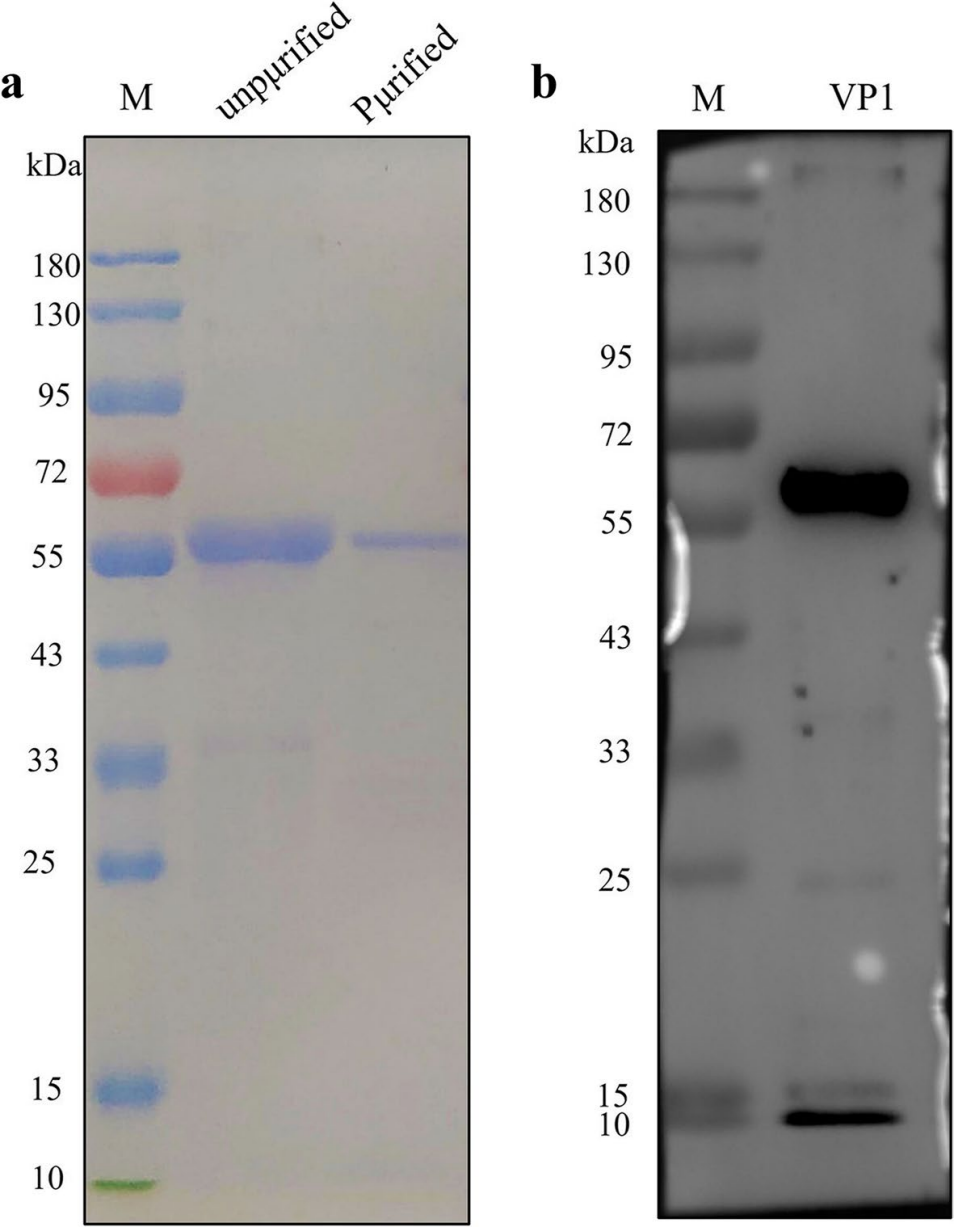
SDS-PAGE and Western blot analysis of GyH1 capsid protein expressed in E. coli. **a** SDS-PAGE results of recombinant GyH1 capsid protein expression in E. coli. M, protein marker; lane 1, prokaryotic expressed capsid protein; lane 2, purified capsid protein. **b** Western blot results of recombinant GyH1 capsid protein. M, protein marker; lane 1, purified GyH1 capsid protein

The pET-32a-VP1 was transformed into competent BL21, and the expressed and purified VP1 (57 kDa) protein was identified by SDS-PAGE (Fig. 1a). The interaction between the recombinant VP1 protein and the anti-GyH1 positive serum was detected by western blot (Fig. 1b).

### Development of indirect ELISA for anti-GyH1 antibody detection

**Fig. 2:**
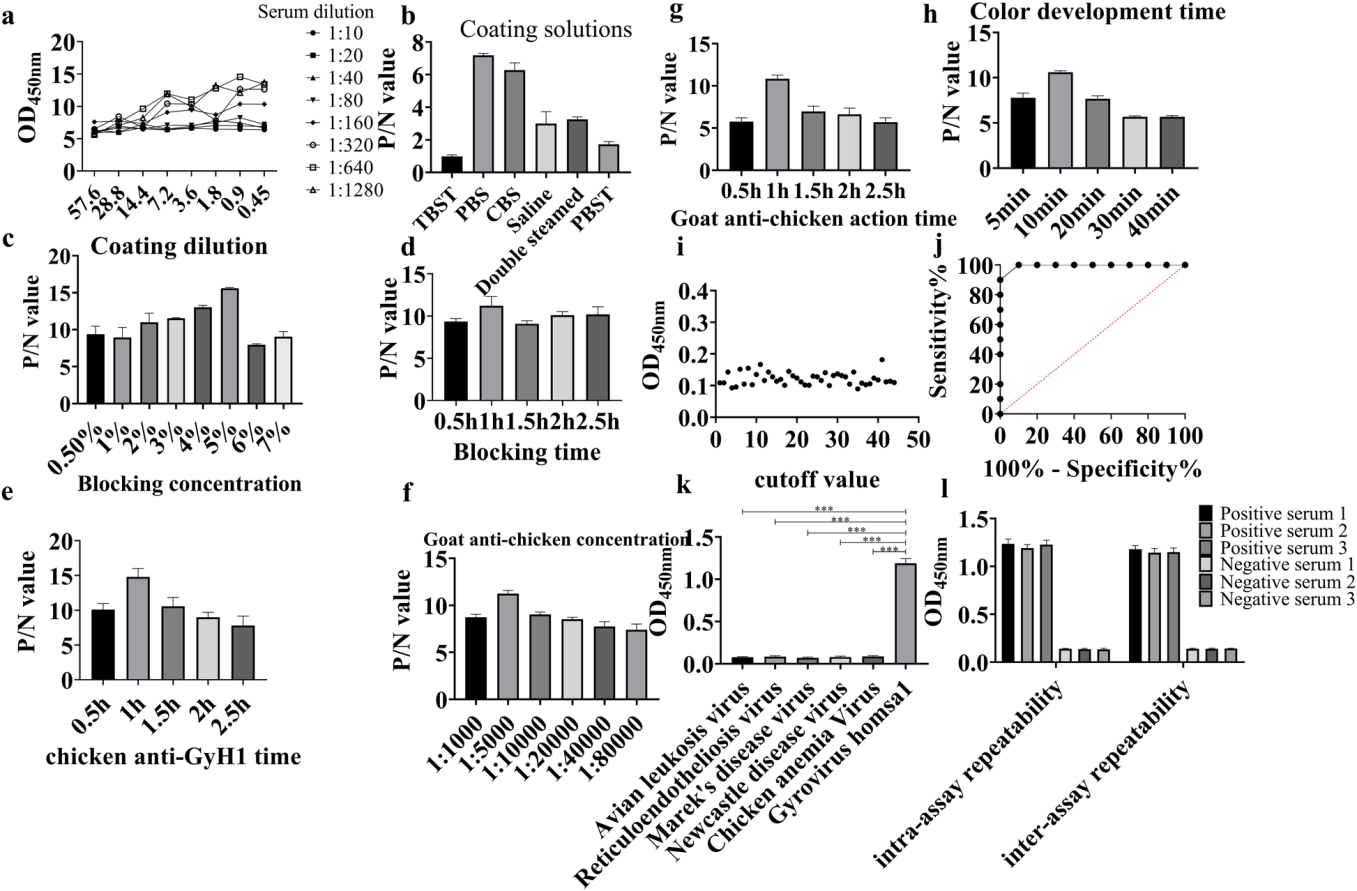
Development of indirect ELISA for anti-GyH1 antibody detection. **a** Optimal concentration of coating antigen and detection antibody. **b** Optimal coating antigen solution. **c** Optimal blocking concentration. **d** Optimal incubation time. **e** Detect the optimal activation time of the antibody. **f** HRP-labeled goat anti-chicken optimal dilution. **g** The optimal activation time of HRP-labeled goat anti-chicken. **h** The best time for color development. **i** ELISA cut-off value. **j** i-ELISA sensitivity test. **k** Specific detection by ELISA. l ELISA repeatability test

To optimize the parameters of antigen coating and serum dilution, a checkerboard titration with different amounts of coating antigen and different serial dilutions of serum were tested. As shown in Fig. 2a, the resulting OD values indicated that the highest P/N ratio was achieved using a concentration of 0.9 μg/mL of coated antigen and a serum dilution of 1:640. 5% skim milk and serum were incubated at 37 °C for 1 h. Using these conditions, the optimal dilution of the secondary antibody was 1:5000, and the optimal incubation time was 1 h at 37 °C. Furthermore, the optimum color change time was 10 min at 37℃.

To determine cutoff values, 42 serum samples from specific pathogen-free chickens were used as negative controls. The resulting OD values showed a mean absorbance of 0.123, with a standard deviation of 0.021. The cutoff value differentiating P/N serum samples were determined as 3 SDs above the mean of the negative controls. Therefore, the threshold of ELISA was 0.123 + 3 × 0.021 = 0.184, and any serum sample with OD_450_ values at or above this cutoff was considered positive. Next, the sensitivity of the GyH1 ELISA was assessed. Starting from a 1:10 dilution in PBS, a twofold serial dilution of GyH1-positive serum samples were analyzed. The maximum dilution that still resulted in a positive value was at a dilution of 1:640, which indicated a high sensitivity (93.4%) of established ELISA. Sera from chickens positive for ALV-J, REV, MDV, NDV and CAV were next assessed for a possible cross-reactivity. As shown in Fig. 2k, all the serum samples had negative results with OD450 values below the cutoff value of 0.184. The above results showed that the established GyH1 ELISA has a desirable specificity with no serological cross-reaction with the chicken pathogens tested. To test the repeatability and reproducibility of ELISA, both GyH1 P/N serum samples were tested with six replicates in six independent ELISA assays. The intra-test CV and the inter-test CV were both lower than 10%, indicating the ELISA has high repeatability and stability (Fig. 2).

### Seroepidemiological survey of GyH1 in China

**Fig. 3:**
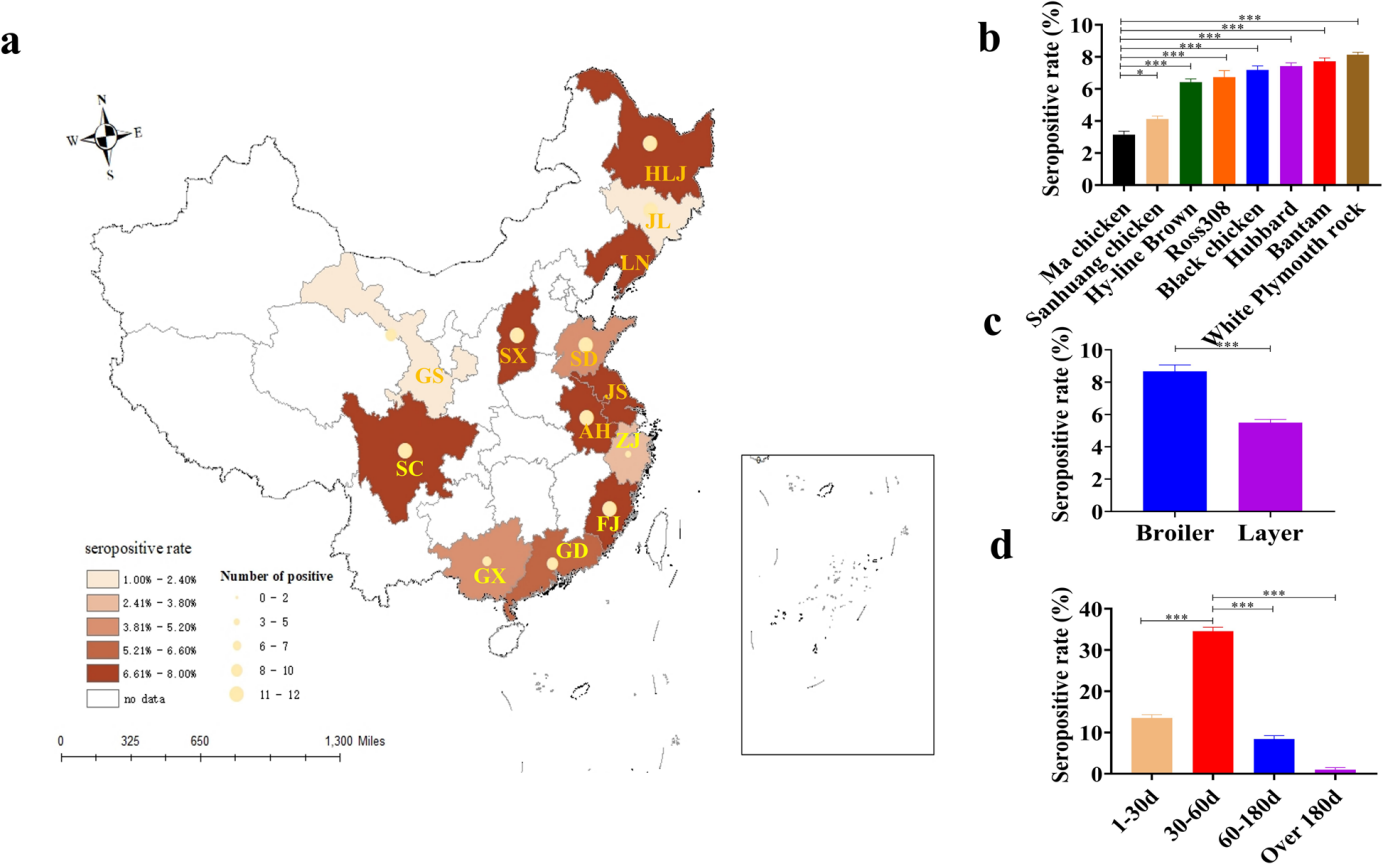
Seroepidemiological investigation of GyH1. **a** Spatial distribution of GyH1 infection in 13 provinces in China. **b** Analysis of susceptibility to GyH1 based on chicken species. **c** Analysis of susceptibility to GyH1 in layers and broiler breeders. **d** Analysis of susceptibility to GyH1 based on age (***: *p* < 0.001; *: *p* < 0.05)

The seroprevalence investigation of GyH1 showed that natural GyH1 infection was widely distributed in 13 provinces in China (Fig. 3a).

The seropositive rate of GyH1 in 13 provinces in China ranged from 0.6% to 7.7% (Fig. 3a). Except for Jilin (JL), Gansu (GS), Zhejiang (ZJ) and Guangxi (GX), there were no significant differences in seropositive rate in other provinces. The seropositive rate of GyH1 in the eight species ranged from 3.1% to 8.1%, with a lower seropositive rate in Chinese native chickens (Fig. 3b). Furthermore, the seropositive rate of GyH1 in broiler breeders and layers was 8.6% and 6.7%, respectively, with a higher seropositive rate in broiler breeders (Fig. 3c). The seropositive rate of GyH1 at 0–30 days, 30–60 days, 60–180 days and over 180 days was 13.5%, 34.4%, 8.3% and 1%, respectively. Chicks had the highest seropositive rate, and the seropositive rate decreased with age (Fig. 3d).

## Discussion

GyH1 can infect chickens, causing aplastic anemia, immunosuppression, and severe damage to multiple systems [18]. Furthermore, Molecular epidemiology showed a GyH1 infection rate of 12.5% in broilers with TVP, indicating that GyH1 infection may be widespread in chickens. Interestingly, it is reasonable to infer that there may be a close association between GyH1 infection and acute childhood diarrhea [15]. To further assess the prevalence of GyH1 infection in commercial chickens, a simple, convenient, high-throughput, high-sensitivity, and specific detection method is urgently needed. Since there is no commercial vaccine against GyH1 in this field, the level of serum antibodies against GyH1 in vivo can reflect the natural infection status of chickens. Therefore, development of highly specific and sensitive ELISA to assess GyH1 infection is necessary.

Serological tests for the detection of virus-specific antibodies are important tools in epidemiological research. However, the application of commonly serological assays, such as virus neutralization assays, enzyme-linked immunosorbent assays, and indirect immunofluorescence assays, often depends on the availability of virus in cell culture. Since GyH1 lacks a cell culture propagation system, PCR has been used as a method to detect GyH1 infection. Since the capsid proteins of some single-stranded circular DNA virus, such as duck circovirus [21], porcine circovirus [22, 23], and chicken anemia virus [24], may induce antibody responses during viral infection, recombinant capsid protein-based serological tests should be useful tool for epidemiological investigations. VP1 is the major capsid protein abundantly produced during GyH1 infection, with high immunogenicity and stability [18–20]. Therefore, we developed an indirect ELISA using the GyH1 viral capsid protein as the coating antigen. ELISA assay can be used to detect specific antibody IgG of all GyH1 strains with sensitivity and specificity of 93.4% and 98.5%, respectively [21–23]. The results showed that the indirect ELISA has high sensitivity and specificity.

A serological survey of GyH1 infection was performed on commercial chicken farms in 13 provinces by applying the developed ELISA under field conditions to develop surveillance and disease control management strategies. Serological surveys showed that serological evidence of GyH1 infection was prevalent in chicken farms in 13 provinces, suggesting that anti-GyH1 antibody in chickens was caused by natural infection. Except for JL, GS, GX and ZJ provinces, there was no significant difference in GyH1 seropositive rate in other provinces. Surprisingly, there was also no significant difference in the number of GyH1 seropositive chickens between most provinces. These data suggest that natural GyH1 infection is naturally prevalent in commercial chickens. In contrast, the lower seroprevalence in JL, ZJ, GX and GS provinces may be due to the random sampling of the survey samples.

A significant correlation between age and susceptibility was observed in many seropositive chickens. The highest seropositive rate in chicks indicates higher susceptibility to GyH1 infection in early life and lower susceptibility to GyH1 infection in later life [25]. Chickens aged 30 to 60 days did not show any clinical signs of GyH1 infection, although they showed high antibody positive after experimental infection with GyH1. We speculate that GyH1 infection may lead to latent infection in asymptomatic chickens, which may be an important risk factor for virus transmission. In this study, no symptoms of GyH1 infection were observed in most chickens, as latently infected chickens may be one of the reasons for the widespread prevalence of GyH1 in China. The correlation analysis of serum OD value of chickens from 1 to 180 days showed that age was weakly negatively correlated with OD value, and the serum OD value of chicks was higher, which means that GyH1 infection may have occurred before serum collection. Correlation analysis was performed on the seropositivity rates of different chicken species, and we observed significant differences in the seropositivity rates of different chicken species, with the lowest seropositivity rate in Chinese native chickens. The possible reasons for this result are that native chickens are mainly free-range, with less chance of exposure to GyH1, or stronger genetic resistance. More than that, poor management of intensive chicken farms may be an important factor in causing GyH1 infection. Next, we counted the seropositivity rates of broiler breeders and layers, and observed that broiler breeders had higher seropositivity than layers, suggesting that broiler breeders are more susceptible to GyH1 infection and that good management can effectively prevent and control GyH1 infection [26–28].

## Conclusion

In this study, an indirect ELISA method was developed for anti-GyH1 antibody detection. In this study, an indirect ELISA method for the detection of anti-GyH1 antibodies was developed. The seroepidemiological results provide evidence for the widespread prevalence of natural GyH1 infection in commercial chickens. Furthermore, latent infection should be considered as a risk factor for widespread and permanent GyH1 transmission in China. Different species have different susceptibility to GyH1, and aged chickens showed obvious age resistance to GyH1. Our study enriches the epidemiology of GyH1 and provides a basis for the prevention and control of GyH1. Further epidemiological surveillance should be undertaken to implement appropriate GyH1 control management.

## Methods

### Virus strain

The SDAU-1 strain of GyH1 (GenBank accession no. MG366592) was stored in the Laboratory of Molecular Pathology of Shandong Agricultural University, China.

### Samples collection

Five chicken farms were randomly selected in each of the 13 provinces in China. A total of 2,055 chicken sera were randomly collected. The serological survey was carried out in Guangdong (GD), Zhejiang (ZJ), Guangxi (GX), Anhui (AH), Shanxi (SX), Jiangsu (JS), Jilin (JL), Liaoning (LN), Heilongjiang (HLJ), Fujian (FJ), Gansu (GS), Sichuan (SC)and Shandong (SD). To demonstrate the sensitivity of species and age to GyH1, we randomly collected different ages and breeds. The chicken species were Ma chicken, San-Huang chicken, Rose 308, Black chicken, Bantam chicken, Hyline Brown, White Plymouth rock, and Hubbard, respectively, and the age was grouped into four categories: 0–30 days, 30–60 days, 60–180 days, and over 180 days. Serum samples were stored at -80℃ before detection.

### Development of indirect ELISA

According to the manufacturer's instructions, a commercial kit (TIANGEN, Beijing, China) was used to extract viral DNA from GyH1-infected tissues. The synthetic gene optimized for expression in E. coli is based on the VP1 sequence. Using *SacI* and *NotI* restriction sites, the sequence code of VP1 sequence was cloned into E. coli expression vector pET32a and expressed in BL21 competent cells. Recombinant protein expression and nickel chelating agarose purification were performed under denaturing conditions. The protein was dialyzed with 0.01 M phosphate buffer pH7.4, and VP1 recombinant protein was detected by SDS-PAGE, and then western blot analysis was performed with chicken anti-GyH1 positive serum.

The newly developed indirect ELISA used checkerboard titration to determine the optimal working dilution of the coated antigen and serum, using a 96-well plate (2.2 IU, COSTAR, USA). Purified VP1 protein was diluted laterally in 96-well ELISA plates by matrix titration. 100 μL of VP1 protein diluted in 0.05 mol/L phosphate buffered saline (pH7.4) with final concentrations of 57.6 μg/mL, 28.8 μg/mL, 14.4 μg/mL, 7.2 μg/mL, 3.6 μg/mL, 1.8 μg/mL, 0.9 μg/mL and 0.45 μg/mL were added to each well and incubated overnight at 4 °C. Then, each well was blocked with 200 μL of 0.5%-7% skimmed milk at 37 °C for 1 h, and washed with phosphate-buffered saline Tween (PBST) for 5 times. Then, 100 μL of 1:10–1:1280 diluted GyH1 serum (positive serum and negative serum) was added to each well, and incubated at 37 °C for 1 h. After washing, they were incubated with 100 μL of HRP-goat anti-chicken immunoglobulin diluted 1:5000–1:80,000 at 37 °C for 1 h. Washed again, and detected with 100 μL of 3,3’,5,5’-tetramethylhydrazine (TMB) at 37 °C for 10 min in the dark. The reaction was then quenched by the addition of 50 μL of 3 M H_2_SO_4_. Optical density (OD) values were measured at 450 nm. Three technical replicates were set for each parameter, and the optimal conditions were obtained by comparing the average positive and negative ratios (P/N) of the parameters. Then the optimal coating solution, optimal blocking concentration, optimal blocking time, optimal serum dilution, optimal serum reaction time, optimal working conditions of enzyme-labeled secondary antibody and optimal color development time were optimized [21].

Through the optimization of the ELISA procedure, the negative serum in 42 chicken sera was selected to determine the cut-off value of the detection. The cutoff value for OD450 to determine virus positivity was calculated according to the following formula: positive and negative cutoff value sample mean (X) + 3 × standard deviation (mean + 3 × SD). If the OD450 value was above the cutoff value, the serum sample was considered positive, otherwise the serum sample was considered negative. To determine the sensitivity of the indirect ELISA, anti-GyH1 positive serum was diluted 1:10, 1:20, 1:40, 1:80, 1:160, 1:320, 1:640, 1:1280 and 1:2560, respectively. Other operating conditions serve as optimal operating procedures.

By anti-Avian leukosis virus subgroup J (ALV-J), anti-Reticuloendotheliosis virus (REV), anti-Marek's disease virus (MDV), anti-Newcastle disease virus (NDV), anti-Chicken anemia virus (CAV) and anti-GyH1 positive sera were tested separately to assess the specificity of indirect ELISA. Six serum samples (3 positives and 3 negatives) were tested on the same assay plate, and each sample was replicated in 6 wells. The intra-plate coefficient of variation (CV) for the same serum sample was calculated to determine the intra-plate repeatability of the test samples. Six different serum samples (3 positives and 3 negatives) were tested with microtiter plates coated at different time intervals. The inter-plate coefficient of variation for the same serum samples was calculated to determine the inter-plate repeatability of the tested samples. The formula for calculating the coefficient of variation within and between batches is as follows: CV = standard deviation (SD)/average OD450(X) × 100% of the samples.

### Statistical analysis

Differences in GyH1 seropositivity among provinces, ages and breeds were analyzed using SPSS software. Sensitivity, specificity, cut-off and reproducibility were analyzed using GraphPad Prism 8. Maps were drawn by Adobe Illustrator CS5. Results were considered significant when *P* values were less than 0.001.

## Supplementary Information

**Additional file 1.** 

## Data Availability

The data supporting the conclusions of this case report are included in this article. All data sets can be requested from correspondence with the authors.

## Abbreviations

ELISA: Enzyme-linked immunosorbent assay
GyH1: Gyrovrius homsa1(also known as Gyrovirus 3)
TVP: Transmissible viral proventriculitis
GyVs: Gyroviruses
ORFs: Open reading frames
REV: Reticuloendotheliosis virus
MDV: Marek's disease virus
ALV-J: Avian leukosis virus subgroup J
NDV: Newcastle disease virus
CAV: Chicken anemia virus
VP1: Variant Protein 1
