# Supplementary figures and images for: Serological investigation of Gyrovirus homsa1 infections in chickens in China
Source: BMC Vet Res. 2022 Jun 18;18:231. doi: 10.1186/s12917-022-03334-0 (PMC9206369; doi:10.1186/s12917-022-03334-0)

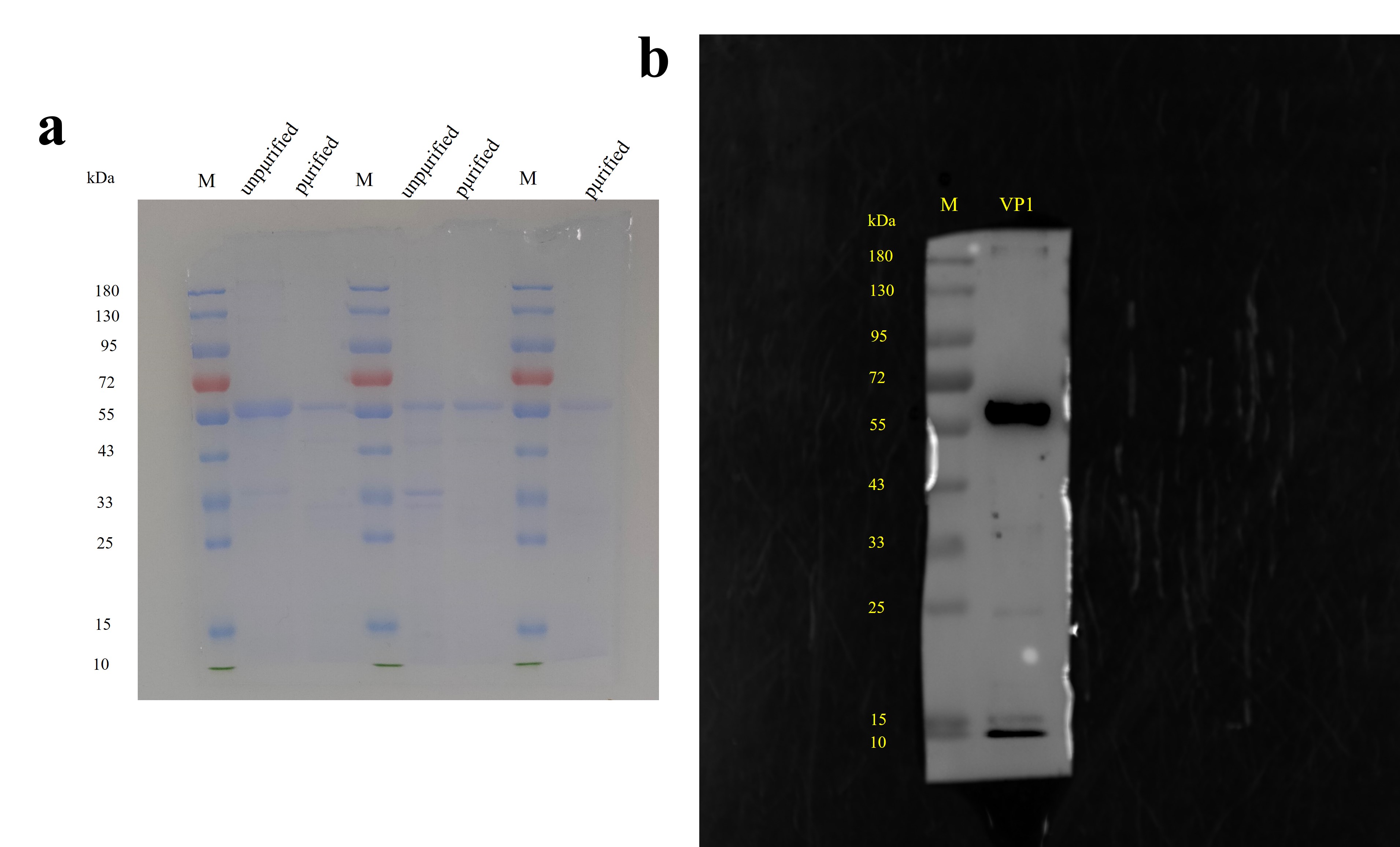

Supplement: Supplementary file 1 — Additional file 1. [file 12917_2022_3334_MOESM1_ESM.jpg]
